# Electronic Devices That Identify Individuals with Fever in Crowded Places: A Prototype

**DOI:** 10.3390/mi8070202

**Published:** 2017-06-24

**Authors:** Carlos Polanco González, Ignacio Islas Vazquez, Jorge Alberto Castañón González, Thomas Buhse, Miguel Arias-Estrada

**Affiliations:** 1 Universidad Nacional Autónoma de México. Av. Universidad 3000 C.P. 04510 Ciudad de México, México; 2 Escuela Superior de Ingeniería Mećanica y Eléctrica. Av. Santa Ana 1000, San Francisco Culhuacan, C.P. 04430 Ciudad de México, México; ignacioisva@gmail.com; 3 Hospital Juárez de México. Av. Instituto Politécnico Nacional 5160, Magdalena de las Salinas, C.P. 07760 Ciudad de México, México; jorge.castanong@gmail.com; 4 Centro de Investigaciones Químicas. Universidad Autónoma del Estado de Morelos. Av. Universidad 1001 C.P. 62209 Cuernavaca, Morelos, México; buhse@uaem.mx; 5 Department of Computer Science, Instituto Nacional de Astrofísica, Óptica y Electrónica. Santa María Tonanzintla 1, 72840 Puebla, México; ariasmo@inaoep.mx

**Keywords:** fever, drone, electronic devices, Arduino platform, translational medicine

## Abstract

Most epidemiological surveillance systems for severe infections with epidemic potential are based on accumulated symptomatic cases in defined geographical areas. Eventually, all cases have to be clinically verified to confirm an outbreak. These patients will present high fever at the early stages of the disease. Here, we introduce a non-invasive low-cost electronic device (bracelet) that measures and reports 24/7, year-round information on the temperature, geographical location, and identification of the subject using the device. The data receiver can be installed in a tower (ground) or a drone (air) in densely populated or remote areas. The prototype was made with low-cost electronic components, and it was tested indoors and outdoors. The prototype shows efficient ground and air connectivity. This electronic device will allow health professionals to monitor the prevalence of fever in a geographical area and to reduce the time span between the presentation of the first cases of a potential outbreak and their medical evaluation by giving an early warning. Field tests of the device, programs, and technical diagrams of the prototype are available as Supplementary Materials.

Fever is a common symptom in infectious processes; however, its form, type, and persistence will vary depending on the disease and clinical context. For example, in cases of Influenza A-H1N1 it will be present in the infected patient during the first hours of the disease [1]; in the case of Zika virus, it will be present after several days. Early detection of febrile symptomatic cases and subsequent etiological identification of the infection are of utmost importance to stop the spread of an outbreak [2]. However, it is unlikely that all symptomatic patients with fever seek medical care, because their symptoms may not be associated with a serious disease or because the patient decides to self-medicate and expects the symptoms subside. A longer time span between the first appearance of symptoms of the infection and the diagnosis and proper treatment of the disease will favor the spread of an outbreak. In order to reduce this time span, a low-cost electronic device has been designed that can be used as a bracelet (data transmitter), giving the body temperature and geographic location of the user, which constitutes an improvement to existing devices owing to its very low cost [3]. The data transmitter works 24/7 and can be used inside homes, hospitals, offices, or outside in stadiums, public transport stations, or shopping malls. This device collects, almost imperceptibly, the temperature, location, and identification of the subject using it. The data receiver for such devices can be installed on a wall or tower (ground-ground mode) or it can be placed on a drone (air-ground mode), in densely populated areas. The manufacture of the transmitter and receiver is not expensive and its electronic parts are easily available, thus its serial production does not present any difficulty. For epidemiological surveillance purposes, the bracelet will be distributed to the population of the community at risk prior to the fly-by of the drone equipped with the receiver; this would provide a time-space record in real time of the prevalence of fever cases in a town or a specific area (“basal” record). The epidemiological surveillance would then focus on the increase of the current vs. the “basal” prevalence of fever cases to give the alert, consequently reducing the time span between the outbreak and the medical evaluation of the affected subjects. In the context of an infectious outbreak already in process, efforts will be focused on monitoring the spread, as well as the effectiveness of the containment measures.

The prototype has two modules: a data transmitter, and a data receiver. Both modules to collect the body temperature and user ID (Figure 1). The transmitter module collects the following data from the subject: identification (Id), body temperature (in degrees Celsius), geographical location (latitude, altitude, longitude), as well as date and recording time (year, month, day, hour, minute) (Table 1). This information is sent to the data receiver module.

The data receiver module receives the information collected by each data transmitter module. This information can be used for any automated epidemiological warning system.

Two data transmitter modules and a data receiver module were tested outdoors (ground-ground mode and ground-air mode) and indoors (ground-ground mode), at peak pedestrian and traffic hours.

Concerning indoor areas only, monitoring was carried out in different areas of a hospital delimited by fixed walls at peak hours of patient traffic (Figure 1). The data receiver module (Figure 2) was fixed to a wall inside the hospital (ground-ground mode), and the data transmitter modules were located on different floors of the hospital (ground-ground mode).

Concerning indoor and outdoor areas, monitoring was carried out in different areas of a hospital and the car park (Figure 1). The data transmitter modules (Figure 2) were placed outdoors (car park) (ground-ground mode), and the data receiver module was placed indoors in the hospital (ground-ground mode).

Concerning outdoors areas only, monitoring was carried out in the vicinity of a stadium (ground-air mode) (Figure 1). The two data transmitter modules (Figure 2) were placed at the sides of the stadium (ground-ground mode), and the data receiver module (Figure 3) was installed on a drone (Figure 1) (mode ground-air), which was flown at a height of 20, 15, and 10 m above the data transmitter modules (Figure 2); they were tested in vertical flight and around the transmitters. A Phantom 1 drone with GPS “lock” was used without camera 4 K and without 3-Axis Gimbal. The weight of the drone could affect its flying time performance, so one solution is to reduce the weight of the data receptor module. Our prototype was reduce from 180 to 65 g, but is possible to reduce it to 30 g without affecting its functions.

No loss of data or connectivity was detected in any of the modes: (i) ground-ground, inside the hospital, outside the hospital, and car park; and (ii) air-ground, crowed place, garden, and car park. The geographical location information (Table 1) was verified by Google Maps for precision; time and temperature were also verified (illustrated by the case of air-ground mode in the garden only). The receptor-transmitter prototype was designed on an Arduino® platform, whose components can be easily assembled at a low cost. The schematic diagrams (Figure 2 and Figure 3) and the programs written in Arduino® software are available in the Supplementary Materials section. This enables the construction of a miniature model to replace the Arduino® platform and reduce the cost of the prototype. The data receiver module had a cost of 40.00 USD, and each data transmitter module has a cost of 40.00 USD (including the GPS tracker) or 5.00 USD (excluding the GPS tracker). In serial production basis, the cost can be reduced 90%. The “granularity” of the information collected by the prototype changes according to the location of the GPS tracker in the modules. If a GPS tracker is located on the data receiver module, the information in the data transmitter module will have the geographical coordinates of the data receiver module location. This will make it difficult to determine the precise geographical location of the subject carrying the data transmitter module (bracelet) and, in turn, it will make it difficult to construct the network of contacts. If a GPS tracker is located on each data transmitter module, the precision of the prototype will make it possible to know the time-space network of contacts of the holder (bracelet) [4,5,6,7,8]. The location of the GPS tracker is closely related to the source of energy of the bracelet, enabling it to use lithium batteries or solar energy converters. The use of this prototype can help in an epidemiological context to reduce the time span between the identification of affected cases and subsequent medical evaluation. It will also provide a continual time-space record, in real time, of the affected population, as well as the immediate identification of the network of contacts of the infected subjects. The adoption of electronic devices among the population implies that it will be offered free of charge, and will thus provide benefits i.e., medical assistance when the electronic device detects a high temperature. A subject with high temperature in a crowed place could become the zero subject of an outbreak. It is necessary to explore other energy sources for this device because, although the electronic device has a negligible cost, the change or recharge of batteries causes discomfort to the end-user. One option is to use piezoelectric mechanisms [9] oriented to recharge the batteries by body movement [10]. The described prototype proved to be a useful tool that can be applied to the horizontal analysis of common symptoms among the population.

The electronic schemes for the device are included (Figure 1 and Figure 2), as well as the software necessary (see Supplementary Materials) to build, improve, or transform this prototype.

## Figures and Tables

**Figure 1:**
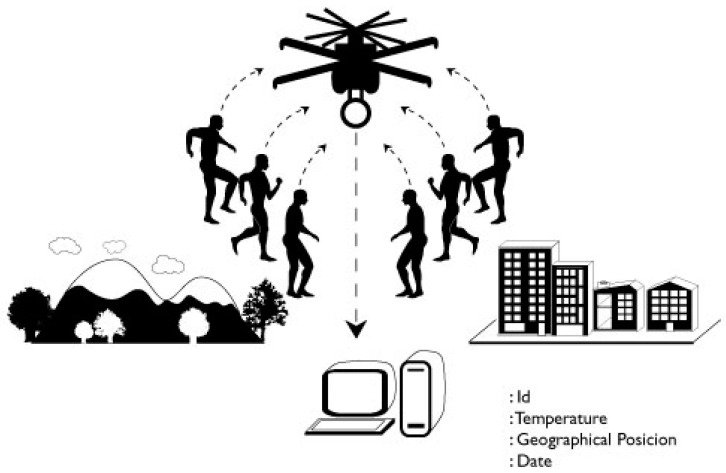
Temperature collection options (air-ground and ground-ground modes).

**Figure 2:**
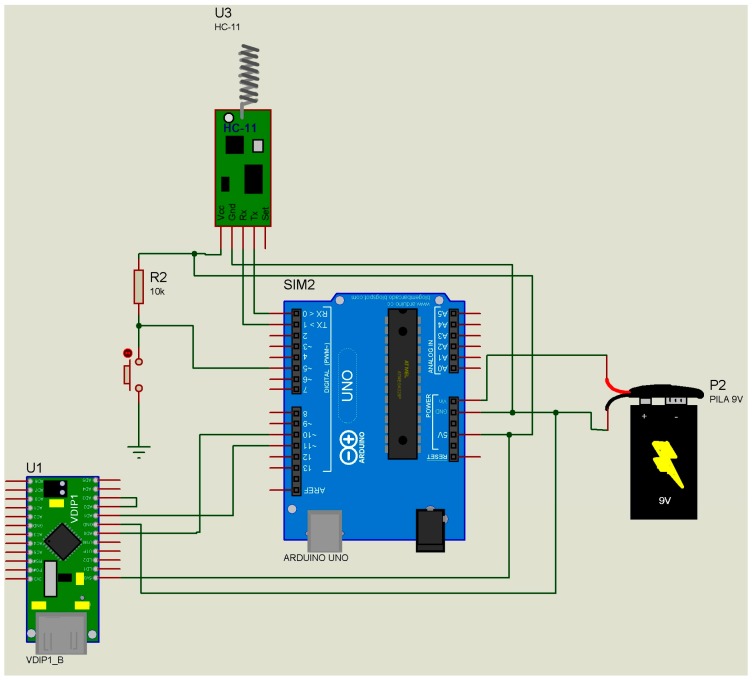
Schematic diagram of the data receiver module.

**Figure 3:**
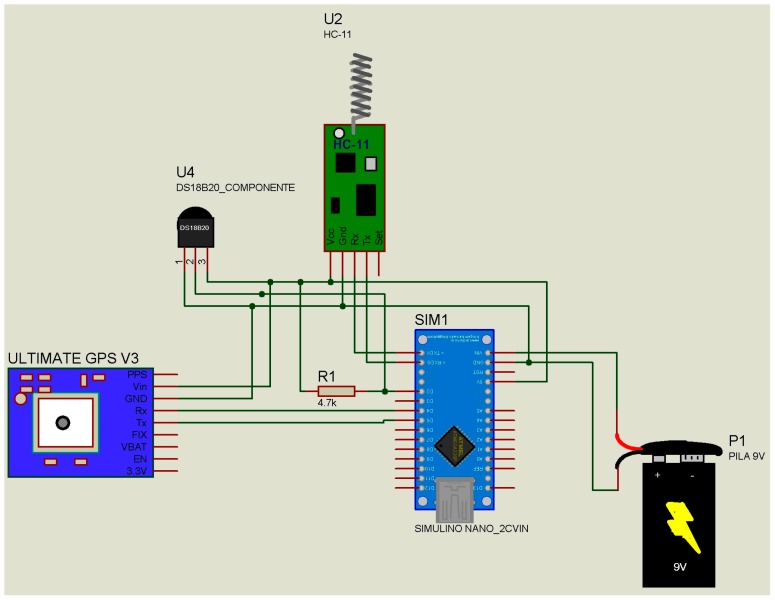
Schematic diagram of the data transmitter module.

**Table 1:** Monitoring data frame.

Start	Id	Temp	Long	Lat	Alt	DD	MM	YYYY	HH	mm	DF	End
@	A	2637	99.1871331	19.333145	86.10	29	06	16	22	19	!	&
@	B	2512	99.1123431	19.966522	02.92	29	06	16	22	20	!	&
@	A	2630	99.1871331	19.333145	86.10	29	06	16	22	20	!	&
@	B	2510	99.1123431	19.966522	02.90	29	06	16	22	20	!	&
@	A	2611	99.1871331	19.333145	86.10	29	06	16	22	20	!	&
@	B	2524	99.1123431	19.966522	02.90	29	06	16	22	21	!	&
@	A	2636	99.1871331	19.333145	86.15	29	06	16	22	21	!	&
@	B	2457	99.1123431	19.966522	02.85	29	06	16	22	21	!	&
@	A	2633	99.1871331	19.333145	86.10	29	06	16	22	21	!	&
@	B	2565	99.1123431	19.966522	02.67	29	06	16	22	21	!	&
@	A	2337	99.1871331	19.333145	72.84	29	06	16	22	22	!	&
@	B	2618	99.1123431	19.966522	02.78	29	06	16	22	22	!	&
@	A	2634	99.1871331	19.333145	86.11	29	06	16	22	22	!	&
@	B	2573	99.1123431	19.966522	02.88	29	06	16	22	22	!	&
@	A	2617	99.1871331	19.333145	86.10	29	06	16	22	23	!	&
@	B	2606	99.1123431	19.966522	02.90	29	06	16	22	23	!	&
@	A	2517	99.1871331	19.333145	86.10	29	06	16	22	23	!	&
@	B	2649	99.1123431	19.966522	02.56	29	06	16	22	23	!	&
@	A	2410	99.1871331	19.333145	86.10	29	06	16	22	23	!	&
@	B	2586	99.1123431	19.966522	02.89	29	06	16	22	24	!	&
@	A	2637	99.1871331	19.333145	85.10	29	06	16	22	24	!	&
@	B	2512	99.1123431	19.966522	02.92	29	06	16	22	24	!	&
@	A	2630	99.1871331	19.333145	86.10	29	06	16	22	24	!	&

Variables collected by the data transmitter breadboard. **Start**: “@” starts recording information. **Id**: Device identification “A, B, C…etc,” this device is associated with the ID information of the bracelet. **Temp**: Temperature in degrees Celsius, e.g., 34.12. **Long**: Geographic longitude. **Lat**: Geographic latitude. **Alt**: Geographic altitude in meters. **DD**: Recording day. **MM**: Recording month. **YYYY**: Recording year. **HH**: Recording hour. **mm**: Recording minutes. **DF**: Dummy filed. **End**: “!” end of the recording.

## Supplementary Materials

The following are available online at www.mdpi.com/2072-666X/8/7/202/s1, Appendix S1: Data receiver prototype; Appendix S2: Data transmitter prototype.
