# Supplementary material for: Electronic Devices That Identify Individuals with Fever in Crowded Places: A Prototype
Source: Micromachines (Basel). 2017 Jun 24;8(7):202. doi: 10.3390/mi8070202 (PMC6189754; doi:10.3390/mi8070202)
Supplement: Supplementary file 1 [file micromachines-08-00202-s001.pdf]

Supplementary Materials: Electronic Devices That Identify Individuals with Fever in Crowded Places: A Prototype

Carlos Polanco González, Ignacio Islas Vazquez, Jorge Alberto Castañón González, Thomas Buhse and Miguel Arias-Estrada

Appendix S1. Data receiver prototype.

```
/*
    Source code: Master
    Project: Temperature
    Carlos Polanco
    Ignacio Islas
    April, 07, 2016     */

#include <SoftwareSerial.h>
SoftwareSerial mySerial(10, 11); // RX, TX

const int buttonPin2 = 2;       // push to send request
const int buttonPin3 = 3;       // push to close the file
const int ledPin     = 13;      // the number of the LED pin

int flag     = 0;
int espera   = 1;

int cero   = 0;
int buttonState2   = 1;         // variable for reading the pushbutton2 status
int buttonState3   = 1;         // variable for reading the pushbutton3 status
int esclavomax      = 1;
int identificador = 1;
int index = 1;

double conta = 170000;

String st_tem = "";

boolean stringComplete = false;

void setup() {
    Serial.begin(9600);
    mySerial.begin(9600);
    pinMode(ledPin, OUTPUT); // initialize the LED pin as an output
    pinMode(buttonPin2, INPUT); // initialize the pushbutton2 pin as an input
```

```
    pinMode(buttonPin3, INPUT);   // initialize the pushbutton3 pin as an input
}

void loop() {
    buttonState2 = digitalRead(buttonPin2);
    buttonState3 = digitalRead(buttonPin3);

    if (stringComplete) {
      if (flag == 0) { // open the USB file
        flag = 1;
        mySerial.print("OPW F040716.TXT\r");
        delay (2000);
      }
      mySerial.print("WRF ");   // write the string in the USB file
      delay (2000);
      mySerial.write(cero);
      mySerial.write(cero);
      mySerial.write(cero);
      mySerial.write(41);
      mySerial.print('\r');
      delay (2000);
      mySerial.print(st_tem);
      mySerial.print("\r\n");
      delay (2000);
      st_tem = "";
      conta = 0;
      stringComplete = false;
    }



    if (conta > 0)   conta--;

      if (buttonState3 == LOW) {
        digitalWrite(ledPin, HIGH);
        delay(2000);
        mySerial.print("CLF F040716.TXT\r"); // close the USB file
        delay (10000);
        digitalWrite(ledPin, LOW);
        index = 9;
      }

    if (conta == 0) {
```

```
            if (index == 1) {
                Serial.print("A");
                conta =    170000;
                index = 2;
            }
            else if (index == 2) {
                Serial.print("B");
                conta =    170000;
                index = 3;
            }
            else if (index == 3) {
                Serial.print("C");
                conta =    170000;
                index = 1;
            }
        }
}


void serialEvent() {
        while (Serial.available()) {
                char inChar = (char)Serial.read(); // get the new byte
                st_tem += inChar; // add it to the st_tem
                if (inChar == '&')    stringComplete = true;    // if the incoming character is a newline, set
a flag, so the main loop can do something about it
        }
}
```

Data receiver Arduino source code.

Appendix S2. Data transmitter prototype.

```
/*
    Source code: Slave
    Project: Temperature
    Carlos Polanco
    Ignacio Islas
    April 07, 2016      */


#include <OneWire.h>
#include <DallasTemperature.h>
#include <Adafruit_GPS.h> // install the Adafruit GPS library
```

```
#include <SoftwareSerial.h> // load the Software Serial library
SoftwareSerial mySerial(5,4); // initialize the Software Serial ports 4 and 5
Adafruit_GPS GPS(&mySerial); //create the GPS object

String inString = "";
String st_tem     = "";      // string to hold input
String NMEA1; // variable for first NMEA sentence
String NMEA2; // variable for second NMEA sentence



int int_temperatura;     // variable to hold temperature
int tem_int;
int v_ascci;

boolean stringComplete = false;   // whether the string is complete

float temperatura = 0.0;
float calculo = 0;

String inputString = "";               // a string to hold incoming data

char inicio ='@';     // start string
char fin ='&'; // end string
char identificador ='A'; // number of slave
char otro   ='!'; // optional field
char dec_t;
char c; //to read characters coming from the GPS



#define ONE_WIRE_BUS 2        // data wire is plugged into port 2 on the Arduino
OneWire oneWire(ONE_WIRE_BUS);       // setup a oneWire instance to communicate with any
OneWire devices (not just Maxim/Dallas temperature ICs)
DallasTemperature sensors(&oneWire);       // pass our oneWire reference to Dallas Temperature



void setup() {
        Serial.begin(9600); // open serial communications and wait for port to open:
        sensors.begin(); // start up the library

        Serial.begin(9600); // turn on serial monitor
        GPS.begin (9600); // turn on the GPS at 9600 bauds
        GPS.sendCommand("$PGCMD,33,0*6D"); // turn off antenna update nuisance data
        GPS.sendCommand("PMTK_SET_NMEA_UPDATE_10HZ"); // set update rate to 10 hz
```

```
    GPS.sendCommand("PMTK_SET_NMEA_OUTPUT_RMCGGA"); // request RMC and GGA
sentences only
    delay (1000);
}

void loop() {

   readGPS();
   delay(12);

   sensors.requestTemperatures(); // Send the command to get temperatures
   if (stringComplete) {
      inputString = "";     // clear the string
      temperatura = sensors.getTempCByIndex(0);   // get the temperature from the module

      st_tem+=inicio;       // to put the start on the string
      st_tem += identificador; // to put id slave on the string

      int_temperatura = temperatura * 100;
      tem_int = int_temperatura / 1000;
      v_ascci = (char)tem_int;
      st_tem+= v_ascci;

      tem_int = int_temperatura - (tem_int * 1000);
      dec_t = tem_int / 100;
      v_ascci = (char)dec_t;
      st_tem+= v_ascci;

      tem_int = tem_int - (dec_t * 100);
      dec_t = tem_int / 10;
      v_ascci = (char)dec_t;
      st_tem+= v_ascci;

      tem_int = tem_int - (dec_t * 10);
      v_ascci = (char)tem_int;
      st_tem+= v_ascci;


      Serial.print(st_tem);

      Serial.print(GPS.longitude,4);
      Serial.print(GPS.latitude,4);
      Serial.print(GPS.altitude);
```

```
        st_tem+= otro;
        st_tem+= fin;     // put end on the string

        st_tem = "";     // clear the string



        stringComplete = false;
    }
}

void serialEvent() {
    while (Serial.available()) {
        char inChar = (char)Serial.read(); // get the new byte
        if (inChar == 'A') stringComplete = true;   // if the incoming character is a newline, set a
flag so the main loop can do something about it:
    }
}



void readGPS() {
    clearGPS();
    while (!GPS.newNMEAreceived()) {   // loop until you have a good NMEA sentence
        c = GPS.read ();
    }

    GPS.parse(GPS.lastNMEA());   // parse that last goo NMEA sentence
    NMEA1= GPS.lastNMEA();

    while (!GPS.newNMEAreceived()) {   // loop until you have a good NMEA sentence
        c = GPS.read ();
    }
    GPS.parse(GPS.lastNMEA());   // parse that last goo NMEA sentence
    NMEA2= GPS.lastNMEA();

 // Serial.println(NMEA1);
 // Serial.println(NMEA2);
 // Serial.println("");

 // Serial.println(GPS.latitude,4);
 // Serial.println(GPS.lat);
```

```
  // Serial.println(GPS.longitude,4);
  // Serial.println(GPS.lon);
  // Serial.println(GPS.altitude);



  //   Serial.print("\nTime: ");
  //   Serial.print(GPS.hour, DEC); Serial.print(':');
  // Serial.print(GPS.minute, DEC); Serial.print(':');
 //   Serial.print(GPS.seconds, DEC); Serial.print('.');
   // Serial.println(GPS.milliseconds);
   // Serial.print("Date: ");
    Serial.print(GPS.day, DEC); //Serial.print('/');
    Serial.print(GPS.month, DEC); //Serial.print("/20");
    Serial.println(GPS.year, DEC);
   // Serial.print("Fix: "); Serial.print((int)GPS.fix);
   // Serial.print(" quality: "); Serial.println((int)GPS.fixquality);
  //   if (GPS.fix) {
  //      Serial.print("Location: ");
   //     Serial.print(GPS.latitude, 4); Serial.print(GPS.lat);
    //   Serial.print(", ");
    //   Serial.print(GPS.longitude, 4); Serial.println(GPS.lon);
   //
    //   Serial.print("Speed (knots): "); Serial.println(GPS.speed);
     // Serial.print("Angle: "); Serial.println(GPS.angle);
    //   Serial.print("Altitude: "); Serial.println(GPS.altitude);
    //   Serial.print("Satellites: "); Serial.println((int)GPS.satellites);
   // }



}




void clearGPS() {
      while (!GPS.newNMEAreceived()) {   // clear old and corrupt data from serial port
            c = GPS.read ();
      }
      GPS.parse(GPS.lastNMEA());   // parse that last goo NMEA sentence
      while (!GPS.newNMEAreceived()) {   // clear old and corrupt data from serial port
            c = GPS.read ();
      }
      GPS.parse(GPS.lastNMEA());   // parse that last goo NMEA sentence
      while (!GPS.newNMEAreceived()) {   // clear old and corrupt data from serial port
```

```
            c = GPS.read ();
      }
      GPS.parse(GPS.lastNMEA());    // parse that last goo NMEA sentence
}
```

Data transmitter Arduino source code.
